# Supplementary material for: Coral fluorescence: a prey-lure in deep habitats
Source: Commun Biol. 2022 Jun 2;5:537. doi: 10.1038/s42003-022-03460-3 (PMC9163160; doi:10.1038/s42003-022-03460-3)
Supplement: Supplementary file 3 — Reporting Summary [file 42003_2022_3460_MOESM3_ESM.pdf]

nature research

Corresponding author(s): Dr. Or Ben-Zvi

Last updated by author(s): Mar 28, 2022

Reporting Summary

Nature Research wishes to improve the reproducibility of the work that we publish. This form provides structure for consistency and transparency in reporting. For further information on Nature Research policies, see our Editorial Policies and the Editorial Policy Checklist.

Statistics

For all statistical analyses, confirm that the following items are present in the figure legend, table legend, main text, or Methods section.

n/a	Confirmed	
☐	☒	The exact sample size (n) for each experimental group/condition, given as a discrete number and unit of measurement
☐	☒	A statement on whether measurements were taken from distinct samples or whether the same sample was measured repeatedly
☐	☒	The statistical test(s) used AND whether they are one- or two-sided<br>*Only common tests should be described solely by name; describe more complex techniques in the Methods section.*
☐	☒	A description of all covariates tested
☐	☒	A description of any assumptions or corrections, such as tests of normality and adjustment for multiple comparisons
☐	☒	A full description of the statistical parameters including central tendency (e.g. means) or other basic estimates (e.g. regression coefficient) AND variation (e.g. standard deviation) or associated estimates of uncertainty (e.g. confidence intervals)
☐	☒	For null hypothesis testing, the test statistic (e.g. F, t, r) with confidence intervals, effect sizes, degrees of freedom and P value noted<br>*Give P values as exact values whenever suitable.*
☒	☐	For Bayesian analysis, information on the choice of priors and Markov chain Monte Carlo settings
☐	☒	For hierarchical and complex designs, identification of the appropriate level for tests and full reporting of outcomes
☐	☒	Estimates of effect sizes (e.g. Cohen's d, Pearson's r), indicating how they were calculated

Our web collection on statistics for biologists contains articles on many of the points above.

Software and code

Policy information about availability of computer code

Data collection	No softwares were used for data collection
Data analysis	All statistical analyses were performed using R software version 3.5.2

For manuscripts utilizing custom algorithms or software that are central to the research but not yet described in published literature, software must be made available to editors and reviewers. We strongly encourage code deposition in a community repository (e.g. GitHub). See the Nature Research guidelines for submitting code & software for further information.

Data

Policy information about availability of data

All manuscripts must include a data availability statement. This statement should provide the following information, where applicable:

- Accession codes, unique identifiers, or web links for publicly available datasets
- A list of figures that have associated raw data
- A description of any restrictions on data availability

The datasets generated and/or analyzed during the current study are available at https://figshare.com/projects/Coral_fluorescence_a_prey-lure_in_deep_habitats_CB/133898

Field-specific reporting

Please select the one below that is the best fit for your research. If you are not sure, read the appropriate sections before making your selection.

☐ Life sciences ☐ Behavioural & social sciences ☒ Ecological, evolutionary & environmental sciences

For a reference copy of the document with all sections, see nature.com/documents/nr-reporting-summary-flat.pdf

Ecological, evolutionary & environmental sciences study design

All studies must disclose on these points even when the disclosure is negative.

Study description	The study includes: (1) ex-situ attraction experiment in which randomly selected individuals of selected taxa were tested for preferential swimming in the presence of fluorescent and non-fluorescent targets, (2) an in-situ attraction experiment in which natural plankton assemblages were tested for preferential swimming in the presence of fluorescent and non-fluorescent targets at sea, and (3) predation experiments in which predation rates of different fluorescence morphs of the coral Euphyllia paradivisa were determined. "color" (which describes the type of target or the fluorescence morph of corals) was tested as a fixed effect while side of the aquarium, coral ID, trial number, position of the target etc were tested as random effects.
Research sample	This study uses Artemia salina, a common crustacean used in many experiments involving corals. A. salina were collected from saline ponds, north of the city of Eilat, Israel. The native crustacean Aniomysis marisrubri is abundant in the reefs of Eilat and was freshly collected prior to the experiments. Sparus aurata fish larvae were obtained from an aquaculture center in Eilat. The organisms were chosen due to their use in marine-related experiments, their visual systems, swimming abilities, relative large size, and the ease of collection and maintenance. The coral Euphyllia paradivisa is an abundant species in the mesophotic reefs of Eilat and displays a variety of fluorescence morphs relevant to this study.
Sampling strategy	While sampling the corals we targeted only the relevant fluorescent morphs keeping a distance of at least 5 m between sampled colonies, however, the specific colonies sampled were chosen randomly. The coral polyps used for the predation experiment were determined using a random ordered list of coral IDs. The plankton individuals used for the ex-situ attraction and predation experiments were chosen randomly.
Data collection	Data resulting from the attraction experiments (in-situ and ex-situ) and the predation experiment were collected manually. The spectral data was recorded on the spectrometer and downloaded to a computer.
Timing and spatial scale	The ex-situ experiments took place over a period of two years (11/2019-07/2021), the in-situ attraction experiment was conducted between March and May 2020, and the predation experiment trials were performed between June and September 2019.
Data exclusions	In order to maintain equal sample sizes between morphs in the predation experiments, the first 42 or 32 trials (for the experiment under blue illumination and red illumination, respectively) were used in the analysis.
Reproducibility	The experiments were repeated multiple times as indicated in the manuscript and methods are detailed to allow reproducibility.
Randomization	In the ex-situ attraction experiment ,the side of the targets was switched between trials in order to assure there is no bias unrelated to the fluorescent or non-fluorescent targets. In our in-situ attraction experiment the position of the trap on the frame (i.e. the position of the trap relative to the surroundings) was changed clockwise at each trial and this was repeated twice (total of 6 trials). In our ex-situ predation experiment an individual coral polyp was not used twice within 24 hours. A list of polyps IDs was generated and polyps were selected in that order.
Blinding	In the ex-situ attraction experiments, the targets were removed after the chamber was divided. Therefore there was no indication for the counter which side contained which target. The same approach was taken in the ex-situ predation experiment when corals were removed from the container prior to the counting of swimming A. salina. For the in-situ attraction experiment, the content of each trap was filtered into a numbered jar with no indication of the trap's color, therefore the counter was blinded for the effect of color.

Did the study involve field work? ☒ Yes ☐ No

Field work, collection and transport

Field conditions	The in-situ experiment was conducted at the depth of 40 m, on sunny days with no-to-mild currents.
Location	Gulf of Eilat/Aqaba, northern Red Sea
Access & import/export	Euphyllia paradivisa colonies were collected at the Dekel Beach at Eilat, Israel (29°32'17''N, 34°56'56''E) at depth of 45 m during an open circuit technical dive. The polyps were detached underwater using a bone cutter and carefully transfered into Ziploc bags. The corals were then transported in containers filled with fresh seawater to an open seawater system at the Interuniversity Institute for Marine Sciences in Eilat and were not sacrificed at the end of the experiment.
Disturbance	To avoid the loss of an entire colony, only few polyps were collected from each colony (allowing the rest of the colony to regenerate). During the in-situ experiment, the frame of traps was carefully placed on a sandy substrate, avoiding any area with live cover.

Reporting for specific materials, systems and methods

We require information from authors about some types of materials, experimental systems and methods used in many studies. Here, indicate whether each material, system or method listed is relevant to your study. If you are not sure if a list item applies to your research, read the appropriate section before selecting a response.

Materials & experimental systems

n/a	Involved in the study
☒	☐ Antibodies
☒	☐ Eukaryotic cell lines
☒	☐ Palaeontology and archaeology
☐	☒ Animals and other organisms
☒	☐ Human research participants
☒	☐ Clinical data
☒	☐ Dual use research of concern

Methods

n/a	Involved in the study
☒	☐ ChIP-seq
☒	☐ Flow cytometry
☒	☐ MRI-based neuroimaging

Animals and other organisms

Policy information about studies involving animals; ARRIVE guidelines recommended for reporting animal research

Laboratory animals	The study did not involve laboratory animals
Wild animals	The scleractinian coral species Euphyllia paradivisa, the crustaceans Artemia salina, and Aniomysis marisrubri and larvae of the fish Sparus aurata were used for the ex-situ attraction and predation experiments. Euphyllia paradivisa colonies were collected at the Dekel Beach at Eilat (29°32'17''N, 34°56'56''E) at depth of 45 m. The polyps were detached underwater using a bone cutter and transfered in Ziploc bags containing fresh seawater to the open seawater system at the Interuniversity Institute for Marine Sciences in Eilat (IUI) under a 12/12 blue light conditions in a open-seawater system. A. salina were collected using a net in saline ponds adjacent to the city of Eilat and transported in containers filled with fresh seawater to the IUI. A. salina were kept for up to 3 days in aerated sea water under ambient light and water were replaced each day. A. marisrubri were collected using a hand-held net and kept in aerated seawater containers at the IUI for a day. S. aurata were obtained from "Ardag" aquaculture facility.
Field-collected samples	see previous section ("Wild animals")
Ethics oversight	This study was conducted under special permit no. 2019/42363 from the Israel Nature and Parks Authority and performed by authorized personal

Note that full information on the approval of the study protocol must also be provided in the manuscript.
