# Supplementary material for: Coral fluorescence: a prey-lure in deep habitats
Source: Commun Biol. 2022 Jun 2;5:537. doi: 10.1038/s42003-022-03460-3 (PMC9163160; doi:10.1038/s42003-022-03460-3)
Supplement: Supplementary file 4 — Description of Additional Supplementary Files [file 42003_2022_3460_MOESM4_ESM.pdf]

Description of Additional Supplementary Files

File name: Supplementary Data 1
Description: Detailed data of the in-situ plankton attraction experiment.
