# Supplementary material for: Coral fluorescence: a prey-lure in deep habitats
Source: Commun Biol. 2022 Jun 2;5:537. doi: 10.1038/s42003-022-03460-3 (PMC9163160; doi:10.1038/s42003-022-03460-3)
Supplement: Supplementary file 5 — Supplementary Information [file 42003_2022_3460_MOESM5_ESM.docx]

**Supplementary Information for**

**Coral fluorescence: a prey-lure in deep habitats**

Or Ben-Zvi, Yoav Lindemann, Gal Eyal, and Yossi Loya

Corresponding author:

Or Ben-Zvi

Email: orbzvi@gmail.com

**This PDF file includes:**

Supplementary Fig. 1

Supplementary Tables 1 and 2


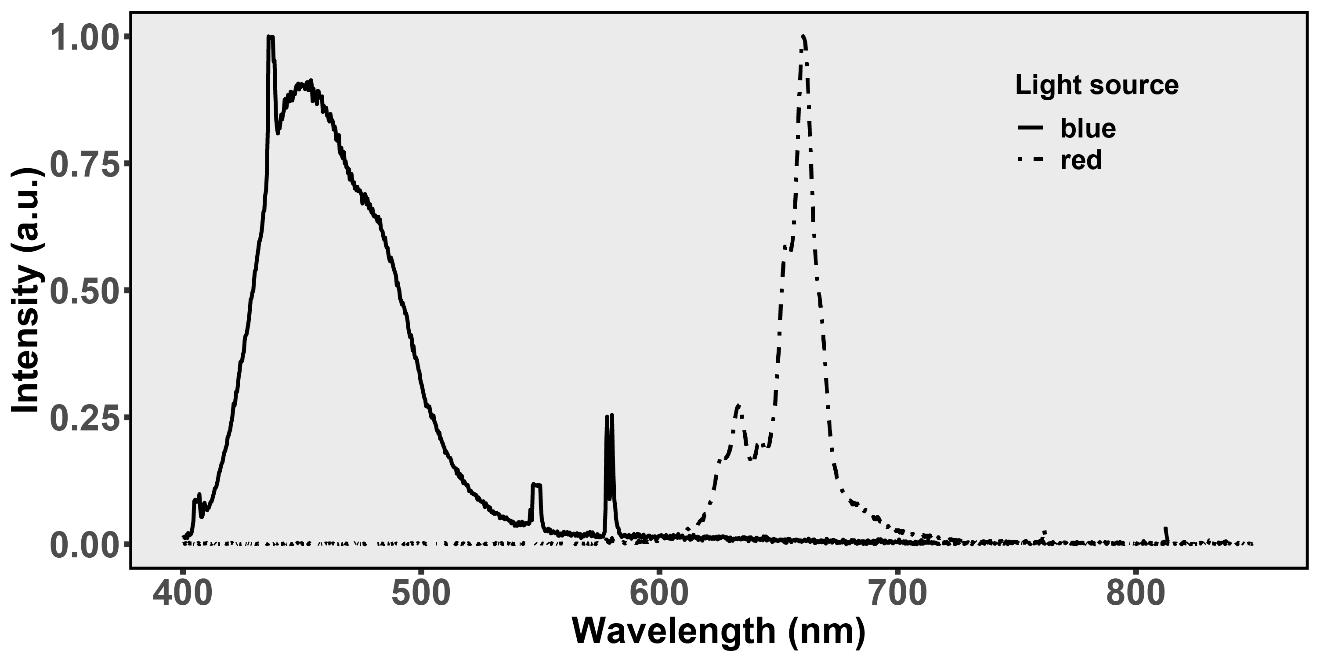
Supplementary Fig. 1: Light spectrum used in the ex situ experiments.

Four blue OSRAM L63W/67 bulbs (i.e., “blue”; solid line) were used for the ex situ experiments. The ex situ predation experiment was repeated also under four red PHILIPS TL-D 36W/15 bulbs (i.e. “red”; dashed line) which does not excite the corals fluorescence. The spectra were measured using a JAZ spectrometer (Ocean Insight, USA) and presented in arbitrary units. The bulbs provided ~30 µmol photons m^-2^ s^-1^.

Supplementary Table 1: Spectral properties of the targets and coral morphs used in the study.

| Sample | Reflectance peak | Fluorescence efficiency | Reflectance yield |
| --- | --- | --- | --- |
| Non-fluorescent green target | 505 nm | NA | 0.31 |
| Non-fluorescent orange target | 595 nm | NA | 0.17 |
| Reflective target | 450 nm | NA | 0.24 |
| Fluorescent green target | 511 nm | 0.315 | NA |
| Fluorescent orange target | 593 nm | 0.320 | NA |
| Clear target (i.e., “control”) | NA | NA | NA |
| Green morph of *E. paradivisa* | 515 nm | 0.526 | NA |
| Yellow morph of *E. paradivisa* | 545 nm | 0.421 | NA |

Supplementary Table 2: Detailed statistical information.

| Experiment | Pair | Test | n | β | SE | Parameter | p-value | Cohen’s d | Model |
| --- | --- | --- | --- | --- | --- | --- | --- | --- | --- |
| Ex situ attraction-Artemia | control-control | GLMM | 10 | -0.02151 | 0.14665 | z=-0.147 | 0.883 | -0.17 | proportion ~ color + (1\|id) |
| Ex situ attraction-Artemia | control- fluorescent green | GLMM | 10 | 1.53 | 0.1606 | z=9.568 | < 2e-16 | 4.13 | proportion ~ color + (1\|side) + (1\|id) |
| Ex situ attraction-Artemia | control- fluorescent orange | GLMM | 10 | 0.68065 | 0.13603 | z=5.004 | 5.62e-07 | 2.03 | proportion ~ color + (1 \| id) |
| Ex situ attraction-Artemia | fluorescent green- fluorescent orange | GLMM | 10 | -0.4394 | 0.1435 | z=-3.061 | 0.0022 | -1.44 | proportion ~ color + (1 \| id) |
| Ex situ attraction-Artemia | green-fluorescent green | GLMM | 10 | -0.6783 | 0.1298 | z=-5.225 | 1.75e-07 | -1.52 | proportion ~ color + (1 \| side) + (1 \| id) |
| Ex situ attraction-Artemia | reflective-fluorescent green | GLMM | 10 | -0.63283 | 0.12452 | z=-5.082 | 3.73e-07 | -2.09 | proportion ~ color + (1 \| id) |
| Ex situ attraction-Artemia | orange-fluorescent orange | GLMM | 10 | -0.75296 | 0.13882 | z=-5.424 | 5.83e-08 | -1.49 | proportion ~ color + (1 \| id) |
| Ex situ attraction-Artemia | reflective -fluorescent orange | GLMM | 10 | -0.70457 | 0.11649 | z=-6.048 | 1.46e-09 | -1.19 | proportion ~ color + (1 \| id) |
| Ex situ attraction-Mysids | reflective-reflective | GLMM | 10 | 0.18404 | 0.15178 | z=1.213 | 0.225 | 0.3 | proportion ~ color + (1 \| id) |
| Ex situ attraction-Mysids | fluorescent green- fluorescent orange | GLMM | 10 | -0.6903 | 0.1615 | z=-4.275 | 1.91e-05 | -1.85 | proportion ~ color + (1 \| id) |
| Ex situ attraction-Mysids | reflective -fluorescent green | GLMM | 10 | -2.2336 | 1.0494 | z=-2.128 | 0.0333 | -1 | proportion ~ color + (1 \| id) |
| Ex situ attraction-Mysids | reflective -fluorescent orange | GLMM | 10 | 0.484 | 0.1458 | z=3.32 | 0.000899 | 1.92 | proportion ~ color + (1 \| id) |
| Ex situ attraction-Mysids | green-fluorescent green | GLMM | 10 | -0.4493 | 0.1657 | z=-2.711 | 0.0067 | -1 | proportion ~ color + (1 \| id) |
| Ex situ attraction-Mysids | orange-fluorescent orange | GLMM | 10 | 0.06061 | 0.1741 | z=0.348 | 0.728 | 0.3 | proportion ~ color + (1 \| id) |
| Ex situ attraction-Fish | reflective-reflective | GLMM | 5 | -0.0381 | 0.27604 | z=-0.138 | 0.89 | 0.04 | proportion ~ color + (1 \| id) |
| Ex situ attraction-Fish | reflective - fluorescent green | GLMM | 5 | -1.9352 | 0.2695 | z=-7.181 | 6.94e-13 | -6.71 | proportion ~ color + (1 \| id) |
| Ex situ attraction-Fish | reflective - fluorescent orange | GLMM | 5 | -1.2793 | 0.2523 | z=-5.071 | 3.96e-07 | -5.52 | proportion ~ color + (1 \| id) |
| Ex situ attraction-Fish | fluorescent green- fluorescent orange | GLMM | 5 | -0.93 | 0.2455 | z=-3.79 | 0.00015 | -2.52 | proportion ~ color + (1 \| id) |
| Ex situ attraction-Fish | green-fluorescent green | GLMM | 5 | 0.08512 | 0.20633 | z=0.413 | 0.68 | -0.27 | proportion ~ color + (1 \| id) |
| Ex situ attraction-Fish | orange -fluorescent orange | GLMM | 5 | -0.2812 | 0.2656 | z=-1.059 | 0.29 | -1.59 | proportion ~ color + (1 \| id) |
| In situ attraction | control | LMM | 6 | 10.09 | 2.707 | t=3.729 | 0.006 | NA | total ~ color + (1 \| run) + (1 \| jar) +(1 \| position) |
| In situ attraction | fluorescent green | LMM | 6 | 5.7 | 2.04 | t=2.8 | 0.02 | 1.88 | total ~ color + (1 \| run) + (1 \| jar) +(1 \| position) |
| In situ attraction | fluorescent orange | LMM | 6 | 5.9 | 2.08 | t=2.84 | 0.02 | 1.68 | total ~ color + (1 \| run) + (1 \| jar) +(1 \| position) |
| Predation | fluorescent green- fluorescent yellow | GLMM | 42 | 0.08317 | 0.03352 | t=2.482 | 0.0131 | -0.46 | count_nsa ~ color + (1 \| color/colony) |
| Predation-control | fluorescent green- fluorescent yellow | LMM | 32 | 0.00348 | 0.234647 | t=0.015 | 1 | 1.47 | count_nsa ~ color + (1 \| color/colony) |
